# The assessment of gestational age: a comparison of different methods from a malaria pregnancy cohort in sub-Saharan Africa

**DOI:** 10.1186/s12884-018-2128-z

**Published:** 2019-01-08

**Authors:** Holger Unger, Kamala Thriemer, Benedikt Ley, Halidou Tinto, Maminata Traoré, Innocent Valea, Harry Tagbor, Gifty Antwi, Prosper Gbekor, Michael Nambozi, Jean-Bertin Bukasa Kabuya, Modest Mulenga, Victor Mwapasa, Gertrude Chapotera, Mwayiwawo Madanitsa, Stephen Rulisa, Maaike de Crop, Yves Claeys, Raffaella Ravinetto, Umberto D’Alessandro

**Affiliations:** 1 Department of Obstetrics and Gynaecology, Simpson Centre for Reproductive Health, Edinburgh Royal Infirmary, Edinburgh, UK; 2 Department of Medicine at the Doherty Institute, The University of Melbourne, Melbourne, Australia; 3 Institute of Tropical Medicine, Antwerp, Belgium; 4 Menzies School of Health Research, Darwin, Australia; 5 Institut de Recherche en Sciences de la Santé - Clinical Trial Unit of Nanoro (IRSS-CRUN), Nanoro, Burkina Faso; 6 School of Medicine, University of Health and Allied Sciences, Hohoe, Ghana; 7 Juaben Government Hospital, Juaben, Ghana; 8 Tropical Diseases Research Center, Ndola, Zambia; 9 Department of Public Health, College of Medicine, Blantyre, Malawi; 10 University of Rwanda, School of Medicine and Pharmacy, Kigali, Rwanda; 11 MRC Unit The Gambia at the London School of Hygiene and Tropical Medicine, London, UK

**Keywords:** Pregnancy, Gestational age, Methods, Ultrasound, Ballard score, Last menstrual period, Symphysio-pubis fundal height, Low income country

## Abstract

**Background:**

Determining gestational age in resource-poor settings is challenging because of limited availability of ultrasound technology and late first presentation to antenatal clinic. Last menstrual period (LMP), symphysio-pubis fundal height (SFH) and Ballard Score (BS) at delivery are therefore often used. We assessed the accuracy of LMP, SFH, and BS to estimate gestational age at delivery and preterm birth compared to ultrasound (US) using a large dataset derived from a randomized controlled trial in pregnant malaria patients in four African countries.

**Methods:**

Mean and median gestational age for US, LMP, SFH and BS were calculated for the entire study population and stratified by country. Correlation coefficients were calculated using Pearson’s rho, and Bland Altman plots were used to calculate mean differences in findings with 95% limit of agreements. Sensitivity, specificity, positive predictive value and negative predictive value were calculated considering US as reference method to identify term and preterm babies.

**Results:**

A total of 1630 women with *P. falciparum* infection and a gestational age > 24 weeks determined by ultrasound at enrolment were included in the analysis. The mean gestational age at delivery using US was 38.7 weeks (95%CI: 38.6–38.8), by LMP, 38.4 weeks (95%CI: 38.0–38.9), by SFH, 38.3 weeks (95%CI: 38.2–38.5), and by BS 38.0 weeks (95%CI: 37.9–38.1) (*p* < 0.001). Correlation between US and any of the other three methods was poor to moderate. Sensitivity and specificity to determine prematurity were 0.63 (95%CI 0.50–0.75) and 0.72 (95%CI, 0.66–0.76) for LMP, 0.80 (95%CI 0.74–0.85) and 0.74 (95%CI 0.72–0.76) for SFH and 0.42 (95%CI 0.35–0.49) and 0.77 (95%CI 0.74–0.79) for BS.

**Conclusions:**

In settings with limited access to ultrasound, and in women who had been treated with *P. falciparum* malaria, SFH may be the most useful antenatal tool to date a pregnancy when women present first in second and third trimester. The Ballard postnatal maturation assessment has a limited role and lacks precision. Improving ultrasound facilities and skills, and early attendance, together with the development of new technologies such as automated image analysis and new postnatal methods to assess gestational age, are essential for the study and management of preterm birth in low-income settings.

**Electronic supplementary material:**

The online version of this article (10.1186/s12884-018-2128-z) contains supplementary material, which is available to authorized users.

## Background

Clinical trials and cohort studies investigating adverse pregnancy outcomes such as preterm birth (PTB, < 37 gestational weeks) and fetal growth restriction (suspected when the birthweight is below the 10th percentile of a birthweight for gestational age standard) rely on fetal biometry to estimate gestational age at delivery [1]. In high-income settings where most women attend health centres early in pregnancy (< 13 weeks) this approach has become routine practice, and pregnancies are dated according to fetal crown-rump length (CRL) [2].

In most low and middle-income countries (LMICs) determining gestational age at rupture of membranes and/or onset of labour is challenging, and postnatally birthweight alone is to crude a measure and is unable to differentiate between growth-restricted and preterm babies [3]. Although ultrasound technology is becoming more affordable and available, access tends to be limited to tertiary centres and private practice; the majority of pregnancies are thus dated using other methods [4]. Last menstrual period (LMP) can predict gestational age well if cycle characteristics and the date of onset of the last menstrual bleed can be clearly established, yet this has proven difficult in many LMIC settings [5, 6]. Symphysio-pubis fundal height (SFH) is a cheap and feasible alternative, appears more accurate than other non-ultrasound based methods, and predicts gestational age at delivery best when sequential measurements are used [5, 7]. SFH measurement at each visit is an essential part of antenatal care and a useful tool to detect pregnancies at risk of adverse outcomes. However, SFH accuracy depends on gestational age and body mass index. [7]

Another option available to healthcare workers in LMICs is the Ballard score (BS) which estimates a gestational age range through postnatal examination of physical and neurological neonatal maturity characteristics [8, 9]. However, its obvious clinical disadvantage is that it cannot be used to instigate critical treatment such as antenatal steroids for fetal lung maturation in women presenting in suspected preterm labour and preterm rupture of membranes before 34 gestational weeks. Nevertheless, it is a practical solution to the aforementioned challenges, in particular when women with no antenatal care come to deliver. Most reports suggest that the postnatal maturation scores are of limited use in LMICs, and perform worse than LMP and SFH [5, 10–13].

Ultrasound is increasingly used in LMICs but its availability remain limited in rural and remote settings; in addition, a great proportion of women still attend late for antenatal care, often after 24 gestational weeks [14]. In the research context, innovative methods are being developed to encourage early presentation [15], but late presentation will remain a critical issue in the general patient population. Using foetal biometry in later pregnancy to estimate gestational age has reduced accuracy as the standard deviation of growth measurements widens and foetal growth aberrations (growth restriction, macrosomia) are more likely. Late pregnancy foetal biometry can be used to correct LMP [11] and, even when used alone, it more accurately predicts gestational age and preterm birth than all other non-ultrasound methods [5], despite the fact that dating by head circumference after 24 gestational weeks (which is the most commonly used measurement) is known to underestimate gestational age, thereby overestimates preterm birth [12].

The present study is a secondary analysis of data collected as part of a large randomised controlled trial to assess the efficacy and safety of four different artemisinin-combination therapies in pregnancy [16]. Using fetal biometry as the reference we assessed the accuracy of LMP, SFH, and BS to estimate gestational age at delivery and preterm birth.

## Methods

This assessment was conducted in the framework of an open label, randomized controlled clinical trial to assess the efficacy and safety of four different artemisinin-combination therapies in women presenting with *P. falciparum* malaria in the second and third trimester of pregnancy. The trial was conducted between June 2010 and August 2013 at seven sites across four countries, namely Burkina Faso, Ghana, Malawi and Zambia (ClinTrial.gov code: NCT00852423). Eligible patients were randomized to one of four treatment arms and followed up weekly until day 63 and then again at delivery. The methods of the trial, including details on quality assurance and quality control are described in detail elsewhere [17], as well as the results of the main outcomes [16].

### Ultrasound

Since only women in the second or third trimester were eligible into the study, gestational age at enrolment was determined using diagnostic ultrasound (US) imaging equipment (FFSonic UF-4100) with a 3.5 MHz transducer for transabdominal examination normally and a 5 MHz transducer for very thin women. Gestational age was calculated based on biparietal diameter, abdominal circumference, and femur length [18] using standard algorithms [19]. For women in the first trimester of pregnancy, the crown-rump length (CRL) was used to confirm exclusion from the study.

Comprehensive quality assurance and quality control (QA/QC) systems were put in place to ensure the quality and reliability of measurements, and the inter-site comparability of US measurements. This included centrally purchased equipment, a standard operating procedure (SOP) which was applicable and mandatory across all sites (Additional file 1), two specifically dedicated staff per site to carry out all US measurements and central training before study start. Periodical training was delivered on site by experienced obstetricians and internal QC measures conducted at each site. This included repeated measurements every first week of the month by the second trained staff member and every third week by repeated measurements of one patient.

### Symphysio-fundal height measurement

SFH measurement was undertaken at enrolment using a non-elastic tape measure. Single measurement was taken from the highest point of the uterus (fundus) to the top of the symphysis pubis.

### Last menstrual period

At enrolment into the study, patients were asked about the date of their LMP. LMP was defined as the date of the first day of the last menstruation.

### Ballard score

The gestational age of babies delivered at the hospital was assessed using the BS. Physical and neurological criteria were recorded according to standard guidelines [8]. Each of the criteria was scored from − 1 to 5. The combined scores range from − 10 to 50, with the corresponding gestational ages being 20 weeks and 44 weeks (2 week range).

### Statistical analyses

All statistical analyses were done using Stata v14 (Stata Corp, USA). For the purpose of this analysis, women with a gestational age > 24 weeks at enrolment, where the birth date of the baby was not documented, who had twins, miscarriages or stillbirths were excluded. In a sensitivity analysis women ≥24 weeks gestational age at enrolment were included.

The level of significance was defined as *p* ≤ 0.05 and US was considered the reference method. Mean and median gestational age for US, LMP, SFH and BS were calculated for the entire study population and stratified by country. Inter-country and inter-method comparisons were done using the Kruskal-Wallis method; correlation coefficients were calculated using Pearson’s rho (r), Bland Altman plots were used to calculate mean differences in findings and 95% limit of agreements (LoA). In order to improve clarity, results of all methods were rounded to the nearest full week for scatterplots [20].

To calculate performance of each method, all babies with gestational age ≥ 37 weeks were categorized as “term”, all other babies as “pre-term”. A “term” result was defined as a negative outcome, a “pre-term” result as a positive result. Sensitivity, specificity, positive predictive value (PPV) and negative predictive value (NPV) were calculated accordingly considering US as reference method [21]. Additional analysis was performed using 32 weeks as a cut-off to define very preterm babies.

## Results

A total of 3428 women in the second and third trimester of pregnancy and with microscopy-confirmed *P. falciparum* infection were included in the main trial [16]. Women with a gestational age > 24 weeks at enrolment (*n* = 1579), those without documented birth date of the baby (*n* = 130) or who had twins (*n* = 38), miscarriages or stillbirths (*n* = 51) were excluded from the present analysis, resulting in a total of 1630 (47.5%) women included. Out of them, 382 (23.4%) were enrolled in Burkina Faso, 582 (35.7%) in Malawi, 294 (18.0%) in Ghana and 372 (22.8%) in Zambia.

The mean maternal age at recruitment was 22.3 years (95%CI: 22.1–22.6), with the lowest mean age of 20.6 years (95%CI: 20.2–21.1) in Zambia and the highest with 24.5 years (95%CI: 23.8–25.2) in Ghana (*p* < 0.001 for all country comparison) (Table 1). There were no differences in mean height and weight by country. Mean gestational age at enrolment was 20.3 weeks (IQR: 16–22) and no patient with gestational age below 13 weeks was enrolled (Table 1).

**Table 1:** Baseline data by country

	All	Burkina Faso	Malawi	Ghana	Zambia	*p*
Mean maternal age at enrollment in years (95%CI)	22.3 (22.1–22.6)	24.0 (23.4–24.5)	21.3 (20.9–21.7)	24.5 (23.8–25.2)	20.6 (20.2–21.1)	< 0.001
Mean maternal weight at enrollment in kg (95%CI)	54.8 (54.4–55.2)	54.6 (53.8–55.3)	54.9 (54.2–55.5)	54.9 (54.0–55.8)	54.9 (53.9–55.8)	0.9
Mean maternal height at enrollment in cm (95%CI)	156.6 (156.3–156.9)	156.6 (156.0–157.2)	156.5 (156.0–157.0)	156.9 (156.1–157.7)	156.6 (155.8–157.3)	0.8
Mean gestational age in weeks at enrolment by US (IQR; range)	20.3 (16–22; 13–24)	20.8 (19–23; 16–24)	19.7 (17–22; 13–24)	20.6 (19–23; 16–24)	20.5 (19–22;16–24)	<0.001
Mean birthweight of baby in gram (95%CI)	2876.7 (2854.1–2900.0)	2844.0 (2800.5–2887.5)	2937.4 (2899.3–2975.6)	2919.8 (2859.4–2980.3)	2780.1 (2734.1–2826.1)	<0.001

Results from BS were available for 93.5% (*n* = 1520) babies, results from SFH for 99.6% (*n* = 1624) mothers and LMP for 24.8% (*n* = 404) of enrolled women.

**Fig. 1:**
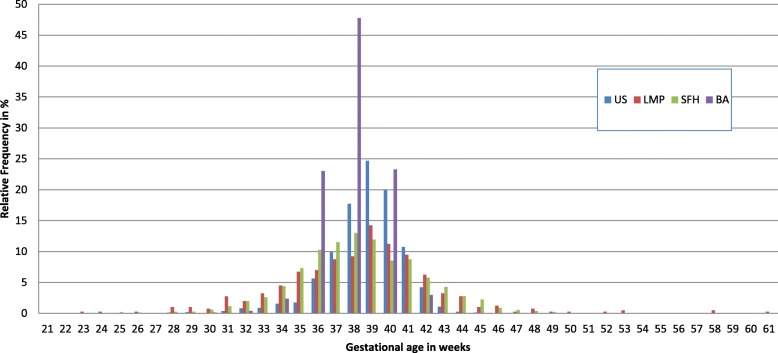
Histogram of the relative frequency of gestational age by US, SFH, LMP and BS

The mean gestational age at delivery using US was 38.7 weeks (95%CI: 38.6–38.8, median: 38.9 weeks, range: 23.1–44.8 weeks), by LMP was 38.4 weeks (95%CI: 38.0–38.9, median: 38.7 weeks, range: 22.9–60.9 weeks), by SFH was 38.3 weeks (95%CI: 38.2–38.5, median: 38.3 weeks, range: 21.1–49.7 weeks) and by BS was 38.0 weeks (95%CI: 37.9–38.1, median: 38.0 weeks, range: 28.0–42.0) (*p* < 0.001) (Fig. 1).

**Fig. 2:**
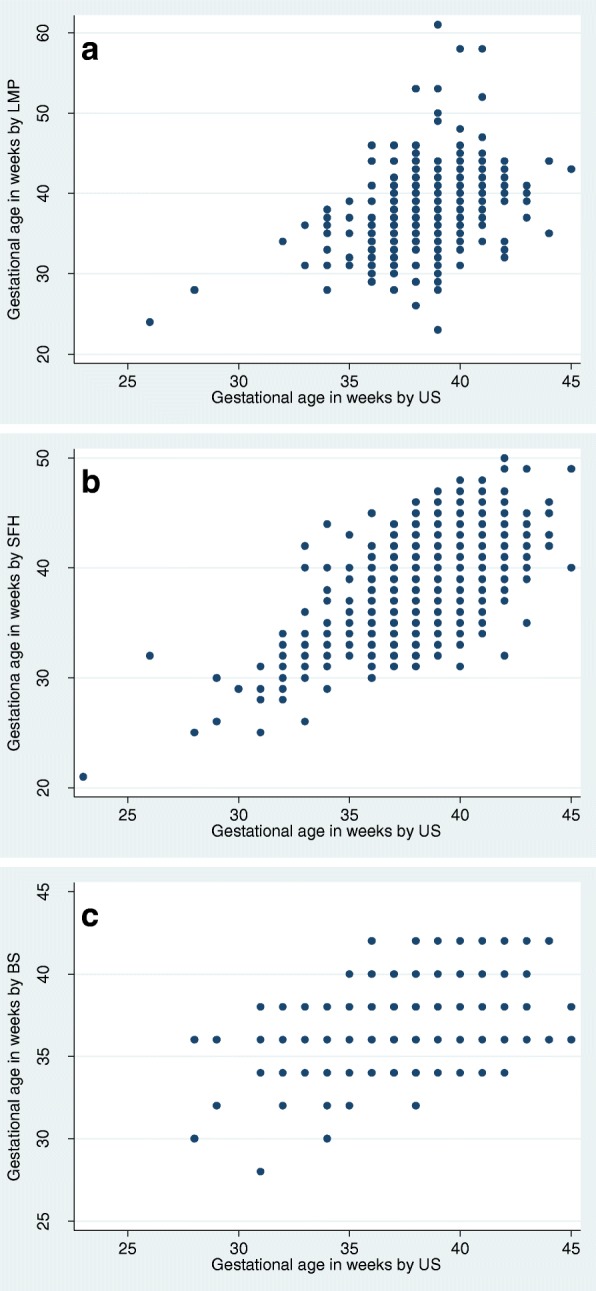
**a**) Scatterplot for LMP with US, **b**)Scatterplot for SHF with US, **c**) Scatterplot for BS with US

Correlation between US and any of the other three methods was poor to moderate (LMP: *r* = 0.38, SFH: *r* = 0.63, BS: *r* = 0.31). However, correlations varied considerably in between countries (Table 2 & Fig. 2).

**Table 2:** Mean gestational age at delivery by different methods and country, Pearson correlation and results from Bland Altman plots

	All	Burkina Faso	Malawi	Ghana	Zambia
Mean gestational age in weeks by US (95%CI)	38.73 (38.63–38.83)	38.85 (38.67–39.03)	38.37 (38.17–38.56)	39.27 (39.06–39.48)	38.74 (38.53–38.96)
Comparison US vs LMP					
N	404	10	30	46	318
Mean gestational age in weeks by LMP (95%CI)	38.41 (37.97–38.86)	40.80 (37.98–43.62)	38.61 (37.27–39.96)	40.26 (39.40–41.11)	38.05 (37.52–38.57)
Pearson r	0.38	−0.06	0.66	0.05	0.38
Mean difference in weeks	0.34	−1.81	−0.24	− 0.96	0.65
95% limit of agreement	−7.9 to 8.6	−9.7 to 6.1	−5.6 to 5.1	−7.28 to 5.36	− 7.99 to 9.28
Range of averages	25.21 to 50.14	37.86 to 44.64	27.93 to 43.00	36.07 to 44.14	25.21 to 50.14
Comparison US vs SFH					
N	1624	382	581	289	372
Mean gestational age in weeks by SFH (95%CI)	38.33 (38.16–38.50)	39.19 (38.91–39.46)	36.18 (35.95–36.41)	38.07 (37.71–38.42)	41.02 (40.69–41.36)
Pearson r	0.63	0.7	0.87	0.54	0.58
Mean difference in weeks	0.4	0.34	2.19	1.23	−2.28
95% limit of agreement	−4.9 to 5.8	−4.2 to 3.5	−5.1 to 4.9	−3.86 to 6.33	−7.56 to 3.00
Range of averages	22.14 to 46.85	29.50 to 44.21	22.14 to 43.64	29.79 to 45.71	29.14 to 46.86
Comparison US vs BS					
N	1538	379	553	242	346
mean gestational age in weeks by BS (95%CI)	38.07 (38.05–38.09)	38.59 (38.55–38.64)	38.47 (38.44–38.50)	38.56 (38.52–38.59)	36.19 (36.16–36.22)
Pearson r	0.31	0.35	0.52	0.45	0.16
Mean difference in weeks	0.8	0.26	−0.03	0.83	2.7
95% limit of agreement	−3.5 to 5.1	−3.6 to 4.1	−3.90 to 3.83	−2.30 to 3.95	−1.21 to 6.62
Range of averages	28.79 to 43.21	29.50 to 42.86	28.78 to 43.21	32.64 to 41.64	32.93 to 40.43

**Fig. 3:**
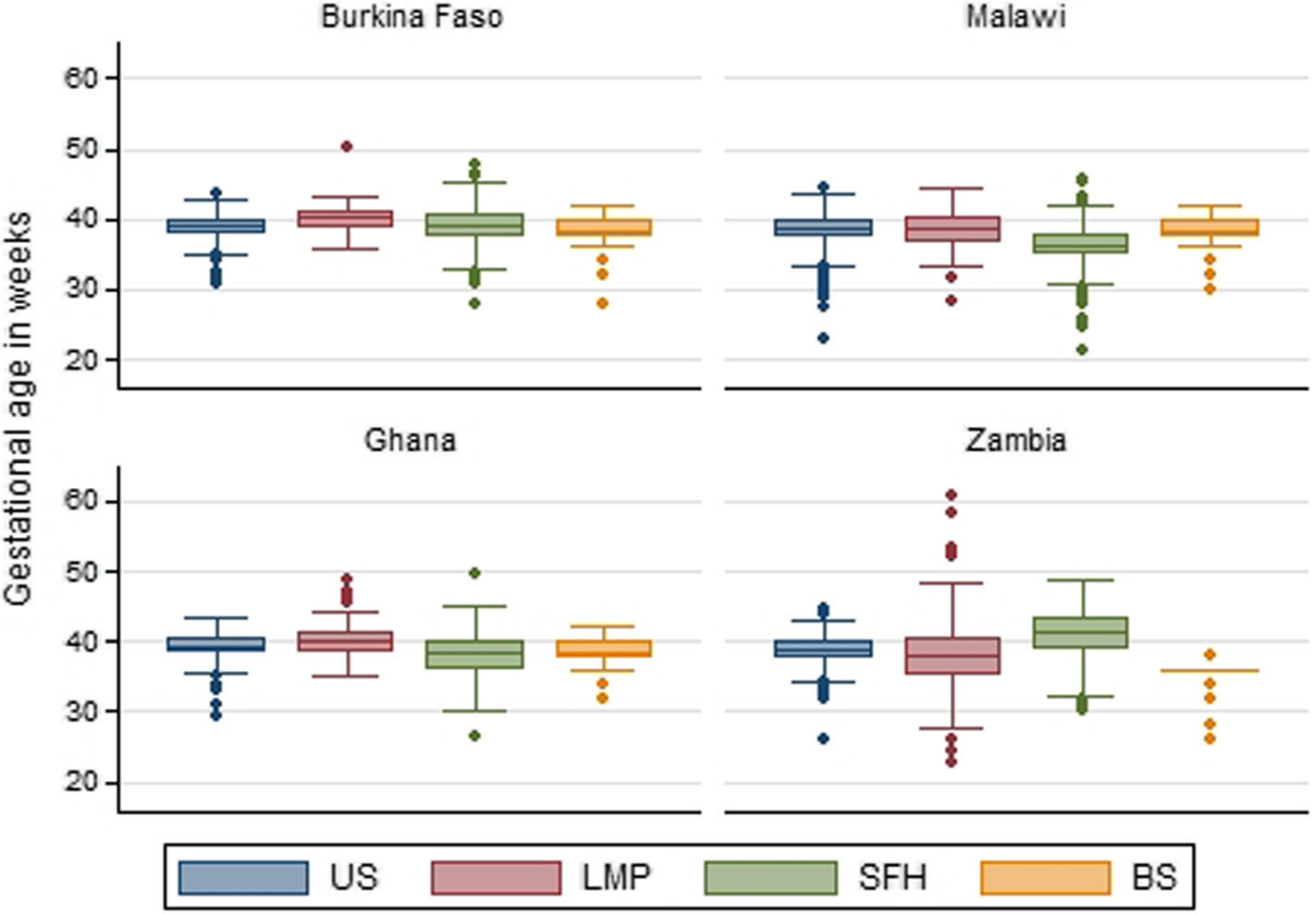
Boxplot of mean gestational age by different methods and country

The mean difference between US and any of the other methods was less than 1 week overall (LMP: 0.34 weeks, SFH: 0.40 weeks, BS: 0.80 weeks) but showed great intercountry variations (Table 2 & Fig. 3). The 95% limits of agreement were considerable (LMP:-7.9 to 8.6 weeks, SFH: − 4.9 to 5.8 weeks, BS: − 3.5 to 5.1 weeks) and again showed great variation in the different countries (Table 2 & Fig. 3).

The sensitivity analyses including women ≥24 weeks’ gestation at enrolment into the study showed results consistent with the main analyses for BS with mean difference of 0.66 weeks (Pearson *r* = 0.27, 95% limit of agreement − 4.1to 5.4, range of averages 27.29–43.21) and higher mean differences for LMP with 1.15 weeks (Pearson *r* = 0.33; 95% limit of agreement − 7.9 to 10.1, range of averages 25.21–50.14 weeks) and SFH with 0.89 weeks (Pearson r = 0.63, 95% limit of agreement − 4.9 to 6.3, range of averages 22.14–46.85 weeks).

Using ultrasound as the reference, 1391 mothers delivered term babies compared to 239 preterm babies (< 37 weeks’ gestation). Sensitivity, specificity, PPV and NPV were 0.63 (95%CI 0.50–0.75), 0.72 (95%CI: 0.66–0.76), 0.28 (95%CI: 0.21–0.36) and 0.92 (95%CI: 0.88–0.95) respectively for LMP, 0.80 (95%CI 0.74–0.85), 0.74 (95%CI 0.72–0.76), 0.35 (95%CI 0.31–0.39) and 0.96 (95%CI 0.94–0.97) respectively for SFH and 0.42 (95%CI 0.35–0.49), 0.77 (95%CI 0.74–0.79), 0.23 (95%CI 00.19–0.27) and 0.89 (95%CI 0.87–0.91) respectively for BS (Tables 3 and 4). Sensitivity across all methods did not differ significantly (*p* = 0.093), whereas all other indicators showed significant differences (all *p* < 0.01). When the cut-off was set at 32 weeks to define very preterm babies sensitivity decreased significantly for LMP and BS to 0.33 (95%CI: 0.01–0.91) and 0.10 (95%CI: 0.001–0.30) respectively (*p* < 0.01 for both), but increased to 0.90 (95%CI: 0.70–0.99) for SFH (*p* < 0.01). Specificity increased to 0.94 (95%CI: 0.91–0.96), 0.98 (95%CI: 0.97–0.98) and 1.00 (95%CI: 1.00–1.00) for LMP, SFH and BS respectively (*p* < 0.01 for all). When comparing true results to false results deriving from the different cut-offs the difference was significant (*p* < 0.0001).

**Table 3:** Extended two-by-two table for dichotomous outcome (term/preterm)

		LMP		SFH		BS	
		≥37 weeks (term)	< 37 weeks (preterm)	≥37 weeks (term)	< 37 weeks (preterm)	≥37 weeks (term)	< 37 weeks (preterm)
US	≥37 weeks (term)	*n* = 246	*n* = 98	*n* = 1025	*n* = 360	*n* = 1001	*n* = 307
	< 37 weeks (preterm)	*n* = 22	n = 38	*n* = 48	*n* = 191	*n* = 123	*n* = 89

**Table 4:** Sensitivity, Specificity, PPV and NPV for different methods to distinguish between term and preterm deliveries (< 37 gestational weeks)

Indicator	LMP	SFH	BS	*p*
Sensitivity (95%CI)	0.63 (0.50–0.75)	0.80 (0.74–0.85)	0.42 (0.35–0.49)	0.093
Specificity (95%CI)	0.72 (0.66–0.76)	0.74 (0.72–0.76)	0.77 (0.74–0.79)	< 0.001
PPV (95%CI)	0.28 (0.21–0.36)	0.35 (0.31–0.39)	0.23 (0.19–0.27)	< 0.001
NPV (95%CI)	0.92 (0.88–0.95)	0.96 (0.94–0.97)	0.89 (0.87–0.91)	< 0.001
Area under the curve	0.67 (0.61–0.74)	0.77 (0.74–0.80)	0.59 (0.56–0.63)	0.0051

## Discussion

Findings of this study suggest that all of three non-sonographic tools to estimate gestational age at delivery generally correlate poor to moderate with US. A single SFH measurement at enrolment (> 13 and < 25 weeks’ gestation) correlates best, and BS the least, with the reference method ultrasound.

The correlation between methods differed substantially depending on country. For example, r was close to 0 (*r* = 0.16) when comparing US and BS in Zambia, however performance was better in Malawi (*r* = 0.5). While a QC/QA system was set up for US, for the other methods such a system was not in place and therefore inter-sites comparability may be limited. When comparing US to LMP the correlation turned negative (*r* = − 0.06) for Burkina Faso and did not excel in any of the other countries. This contradictory finding may partly be due to the low sample size for Burkina Faso for LMP data, but presumably also reflects the substantial recall bias and uncertainty due to irregular menstrual period as well as potential differences in literacy rates.

In settings with limited access to ultrasound, SFH may be the most useful antenatal tool to date a pregnancy, at least at the range of gestational age at enrolment in this study. This is corroborated by findings from previous research, and precision may be improved when multiple measurements are available [5, 7]. Although there were differences in correlation across country sites, these were less marked for SFH than for BS.

In a research context in LMIC settings, ultrasound dating and early attendance are pivotal to assess outcomes such as gestational age at delivery and preterm birth correctly. Yet, since such equipment and expertise are often unavailable SFH (preferably sequential) is probably the best alternative to US measurements in routine care and for further clinical management of the pregnancy. In order to ensure quality measurements, healthcare workers must be taught to assess SFH in a methodical manner [22], and should be supported by ongoing training and audit.

This paper provides further confirmation of the limitations of BS postnatal maturation assessment for pregnancy dating. One significant issue will undoubtedly be that of training and may explain the poor correlation observed at the site in Zambia; BS assessments, in particular its neurological component require training and refresher training [23]. Postnatal maturation assessment is the most complex of all gestational age estimation methods, whereby the examiner is required to adequately assess and process ‘images’ and findings from clinical examination. When one or more SFH measurements are available it may be reasonable to forgo BS assessments and focus limited resources and time on other assessments and activities, such as effective neonatal resuscitation and accurate birthweight measurements. BS retains a role in unbooked pregnancies, and its predictive ability in this context may be improved by taking birthweight into account [24], and by establishing and evaluating quality control and training methods for use in busy clinical settings. Improving the postnatal prediction of gestational age is subject of an ongoing large multi-centre study, which aims to develop a simplified and pragmatic algorithm based on existing assessment approaches, anthopometry and neonatal feeding maturity [25].

A recent call by the Bill and Melinda Gates Foundation has recognised the need for new postnatal tools [26]. Approaches such as using newborn infant screening metabolite measurements [27], complex modelling integrating a number of simple clinical parameters [25], smartphone ultrasound devices, and automated image analysis are being evaluated at present.

Accuracy of any method, including ultrasound, is assessor-dependent; training and quality control are key tools to ensure optimal measurements, which can be achieved for routine care in challenging LMIC settings [28]. Expanding ultrasound services in low-income settings may be a key strategy to improve pregnancy care and outcomes [29], and is certainly feasible [28, 30]. Handheld ultrasound devices, including smartphone ultrasound, may assist with expanding services in LMICs, and batteries can be charged using solar power.

The present evaluation has a number of limitations. The current reference standard for pregnancy dating is the measurement of the fetal crown-rump length before 13 weeks’ gestation. No measurements were done at this early gestation due to the inclusion and exclusion criteria of the main trial, and thus algorithms estimating gestational age from fetal head circumference, femur length and abdominal circumference had to be used, introducing imprecision [12]. Amongst women > 24 weeks gestational age at enrolment the sensitivity analyses showed no major difference in trends with regards to agreement between methods for BS but showed higher mean difference in weeks for SFH suggesting an increasing variation [12]. Moreover, analyses were performed on measurements taken amongst women with malaria infection, which may cause early fetal growth restriction [31], and could lead to an underestimation of gestational age, in particular when HC and FL are used to date the pregnancy.

Lastly, data analysis for LMP was limited primarily to one site only, and only one SFH per women was available for analysis. However, the sample size for other measurements was adequate.

## Conclusions

In conclusion, in settings where ultrasound scanning is still limited SFH may be the most useful tool to predict gestational age at delivery if measured between 13 and 24 gestational weeks amongst women undergoing treatment for *P. falciparum* malaria. Postnatal maturation assessments have a limited role and lack precision. Improving ultrasound facilities and early attendance, together with the development of new technologies such as automated image and video analysis for both ultrasound and BS and new postnatal methods to assess gestational age, will greatly assist with the management of preterm birth in low-income settings.

## Additional files

Additional file 1: Standard operating procedures (PREGACT study) for obstetric ultrasound for the assessment of embryos and foetuses during pregnancy. (PDF 165 kb)

## Abbreviations

BS: Ballard score
CRL: Crown-rump length
LMCIs: Low and middle-income countries
LMP: Last menstrual period
NPV: Negative predictive value
PPV: Positive predictive value
PTB: Preterm birth
QA/QC: Quality assurance and quality control.
SFH: Symphysio-pubis fundal height
SOP: Standard operating procedure
US: Ultrasound
