# Supplementary material for: The assessment of gestational age: a comparison of different methods from a malaria pregnancy cohort in sub-Saharan Africa
Source: BMC Pregnancy Childbirth. 2019 Jan 8;19:12. doi: 10.1186/s12884-018-2128-z (PMC6323786; doi:10.1186/s12884-018-2128-z)
Supplement: Supplementary file 1 — Standard operating procedures (PREGACT study) for obstetric ultrasound for the assessment of embryos and foetuses during pregnancy. (PDF 165 kb) [file 12884_2018_2128_MOESM1_ESM.pdf]

Title: Obstetric ultrasound for the assessment of embryos and foetuses during pregnancy.

1. General Information

1.1 Aim and application:
Obstetric ultrasound will be used for the examination of foetal development upon enrolment into the study in order to make sure that pregnant women for less than 16 weeks are excluded.

Ultrasound in the context of this study will be used to:
- estimate the gestational age and expected delivery date;
- check whether the foetus(es)/embryo(s) is/are alive;
- recognize single or multiple pregnancies;
- locate the pregnancy (intrauterine, ectopic) and the placenta (right, midline, left; anterior, fundal, posterior; central or marginal placenta praevia).

This assessment is intended to complement the study of the efficacy of artemisinin-based combination treatments in pregnancy.

1.2 Principle

Keep in mind that the accuracy of gestational age assessment by ultrasound is greater when the assessment is done earlier in pregnancy.
A rough thumb-rule:

Timing of examination	Precision
6-12 weeks	+/- 3 days
13-20 weeks	+/- 1 week
21-24 weeks	+/- 10 days
25-28 weeks	+/- 2 weeks
> 28 weeks	+/- 3 weeks

1.3 Reagents
NA

1.4 Apparatus, equipment and materials
- Diagnostic Ultrasound Imaging Equipment (FFSonic UF-4100)
- Acoustic coupling agent (ultrasound gel)
- Ultrasound register
- Source document

1.5 Sample for analysis
NA

2 Responsibilities

[…]

3 Definitions and abbreviations:

AC abdominal circumference
BPD biparietal diameter
CRL crown rump length
HC head circumference
FHR foetal heart rate
FL femur length
GS gestational sac
LMP last menstrual period
PW pregnancy week
US ultrasound

4 Method:

4.1 Starting the ultrasound machine:

At first use, set date, time and study site/hospital name.
Press the MENU key to display the MENU PAGE 1. Select "ENTER" by turning the CONTROL knob and press the SET key. Select "DATE" and press the SET key to come to the "DATE TIME" display.
Select "DATE" to set the current date and "TIME" to set the time.
Select "D-FORM" and press the SET key until the date appears in European format (dd-mm-yy).
Select "T-FORM" and press the SET key until time appears without second indication (hh:mm).
Select "EXIT" and press SET to exit the date and time setting menu.

Select "HOSPIT" by turning the CONTROL knob and press the SET key. The character cursor (_) is displayed at the head of the hospital name display area. Use the keyboard to input PREGACT followed by the name of your study site and/or hospital.
Select "ENTER" and press the SET key to register the hospital name.
Select "EXIT" and press SET to exit the hospital name input menu.

Press the MENU key to clear the menu.

Before obstetrical measurement, be sure to register the measurement tables for BPD, CRL, FL, AC and HC according to the <American/European system> using the OB CALC key on the operation panel.

4.2 Examining a new patient

Switch on the echography machine.
Before starting, make sure that the date and the time are displayed correctly on the screen.

Press the ID ? key to input ID (screening No, initials, etc.) using the keyboard.
After input, press ID ? key once again.

If information is available, enter the first day of the last normal menstrual period (LMP).
Press the OB CALC key to display the FUNCTION MENU. Select the “G-CALC” by turning the CONTROL knob and press the SET key. In the following menu, move the cursor to “LMP” and press the SET key again.
You can now enter the date of the last menstrual period (dd/mm) using the numeric keys on the keyboard. Select “ENTER” and press the SET key. The current gestational weeks (in number of completed weeks and days) and the expected date of confinement (dd.mm.yy) are calculated automatically and will now be displayed on the screen below the LMP.

4.3 Preparation of the patient

- Explain to the patient what you will be doing.
- The patient should be lying comfortably on her back, the head slightly raised. Apply acoustic coupling agent liberally to the lower abdomen.
- Use 3.5 MHz transducer for transabdominal examination normally and a 5 MHz transducer for very thin women.
- Position the transducer longitudinally over the lower abdomen with the orientation mark pointing towards you or upwards (to the head of patient).
- Adjust the gain and focus to produce the best image.
- Once you are satisfied with the image quality, start the obstetric measurements.

4.4 Obstetric measurements

- ✓ Look for the foetal heart beat to confirm viability.
- ✓ Check for multiple pregnancy (assess the number of gestational sacs, embryos or heart beats).
- ✓ To measure the biparietal diameter (**BPD**) look for the foetal head and try to centralise it such that you don`t see eye orbits or the back of the head (cervical bones) and a straight line will be apparent.
- ✓ To measure the abdominal circumference (**AC**) make sure you don`t have the heart in the centre, no ribs are visible (but just a point through them) you may see stomach, bladder and umbilical vein
- ✓ Measure femur length (**FL**), make sure the whole femur is visible, if you see more than one, measure the one more close to the surface
- ✓ If the woman is in the first pregnancy trimester, measure the crown-rump length (**CRL**) (head to tail)
- ✓ Wipe off rests of ultrasound gel from the patients abdomen and ask her to clean any remaining rests herself. Tell the patient what you have found and reassure her that her baby is doing well.
- ✓ After having finished all measurements, press the Z key to display a list of all measurements. Enter additional comments by pressing the COMMENT key and moving the cursor with the TRACKBALL.
- ✓ Print out a copy of the report for the patient file.
- ✓ Press the Z key to end the report display.

NOTE! Report contents will be deleted by pressing the NEW PATIENT key, by changing the ID or by turning the power off.

5 Quality control

Every first week of the month, ask a colleague to re-examine the patients seen for ultrasound on any of the follow-up visits at Day 1 to 7.
Every third week of the month, re-examine yourself the patients on any of the follow-up visits at Day 1 to 7.

6 Technical validation

NA

7 Recording and interpretation of results

Heart motion is perceptible from about 6 weeks onwards.
The crown-rump length (CRL) is the most reliable parameter for estimating gestational age up to the eleventh week. From the twelfth week onwards, the biparietal diameter (BPD) is more accurate. After the second trimester, precision of gestational age estimation can vary +/- 3 weeks.

8 Clinical validation/release of results

NA

9 Reporting

If printer is available, print two scans of the foetus, one for the patient and one for documentation (e.g. year/date/time and display information) for the study using the printer (FVP-800) and the summary report. If printer is not available, copy the summary report onto the source document.

10 Storage of samples

NA

11 Training

NA

12 Method validation and literature

Manual of diagnostic ultrasound / edited by P. E. S. Palmer.
World Health Organization 1995

FF sonic UF-4100/UF-4100A
Diagnostic ultrasound imaging equipment
Operation Manual

Fukuda Denshi 2002

13 Comments

14 Safety and environment

There is no significant evidence that US is harmful to human beings, but it is advised that examination using US be kept to a minimum period of time. The probe should be removed from the abdomen whenever the examination is interrupted. Avoid unnecessary use of Doppler.

15 Attachments and forms for completion

16 Revision

**Revision**	
**Changes with respect to the previous published version:**	**A concise overview of the changes made and if possible, refer to documents or observations which form the basis of the changes made.<br>E.g. `chapter 4.3 was adapted on the basis of change control form… - 09-001.<br>E.g. `chapter 4.1 was completed as a result of external audit observation B1.**

17 Approval and distribution

**Approval and distribution**	
	**Name and function**
**Initiated by:**	Sabine Gies<br>Stephen Rulisa
**Approved by:**	Name and function of the person(s) approving the document
**Manual distribution:**	Indicate the manual distribution of the document.<br>E.g. 'No manual distribution.'<br>E.g. '1 copy available in the laboratory.'<br>Preferably no hard copies of this document should be made unless absolutely necessary.
